# A review on the importance of miRNA-135 in human diseases

**DOI:** 10.3389/fgene.2022.973585

**Published:** 2022-09-06

**Authors:** Sepideh Kadkhoda, Solat Eslami, Bashdar Mahmud Hussen, Soudeh Ghafouri-Fard

**Affiliations:** ^1^ Department of Medical Genetics, School of Medicine, Tehran University of Medical Sciences, Tehran, Iran; ^2^ Dietary Supplements and Probiotic Research Center, Alborz University of Medical Sciences, Karaj, Iran; ^3^ Department of Medical Biotechnology, School of Medicine, Alborz University of Medical Sciences, Karaj, Iran; ^4^ Department of Pharmacognosy, College of Pharmacy, Hawler Medical University, Erbil, Iraq; ^5^ Center of Research and Strategic Studies, Lebanese French University, Erbil, Iraq; ^6^ Department of Medical Genetics, Shahid Beheshti University of Medical Sciences, Tehran, Iran

**Keywords:** miRNA, miR-135, cancer, expression, biomarker

## Abstract

MicroRNA-135 (miR-135) is a microRNA which is involved in the pathoetiology of several neoplastic and non-neoplastic conditions. Both tumor suppressor and oncogenic roles have been reported for this miRNA. Studies in prostate, renal, gallbladder and nasopharyngeal cancers as well as glioma have shown down-regulation of miR-135 in cancerous tissues compared with controls. These studies have also shown the impact of miR-135 down-regulation on enhancement of cell proliferation and aggressive behavior. Meanwhile, miR-135 has been shown to be up-regulated in bladder, oral, colorectal and liver cancers. Studies in breast, gastric, lung and pancreatic cancers as well as head and neck squamous cell carcinoma have reported dual roles for miR-135. Dysregulation of miR-135 has also been noted in various non-neoplastic conditions such as Alzheimer’s disease, atherosclerosis, depression, diabetes, Parkinson, pulmonary arterial hypertension, nephrotic syndrome, endometriosis, epilepsy and allergic conditions. In the current review, we summarize the role of miR-135 in the carcinogenesis as well as development of other disorders.

## Introduction

MicroRNAs (miRNAs) represent a group of small-sized transcripts with high impact on the regulation of gene expression. For many years the role of miRNA is mostly associated with translation arrest. miRNAs have crucial roles in the developmental processes and have various biological functions (Fu et al., 2013; Ghafouri-Fard et al., 2021a; Hussen et al., 2021). The process of miRNA synthesis contains multiple steps with the final step being incorporation of either the 5p or 3p strands of the mature miRNA duplex into a complex, namely miRNA-induced silencing complex (miRISC). Notably, shuttling of this complex inside the cell has an essential role in the extent of miRNA-mediated modulation of gene expression (O'Brien et al., 2018; Ghafouri-Fard et al., 2021b; Ghafouri-Fard et al., 2021c; Ghafouri-Fard et al., 2021d). miRNAs regulate gene expression in a dynamic manner which results in buffering of expression levels to reach a stable state (O'Brien et al., 2018). Most remarkably, accessibility and relative abundance of miRNAs and their targets can define the genes that are modulated by miRNAs. Moreover, miRNA-mediated inhibition of target transcripts is not universal among different kinds of cells. This effect is modulated by alternative splicing/polyadenylation events and the amounts of cell type-specific factors that change secondary structure of target transcripts (O'Brien et al., 2018).

The impact of several miRNAs in normal developmental and pathological conditions has been investigated unraveling a wide variety of different functions for these transcripts. Most notably, circulating miRNAs have recently attracted attention of researchers for their application in diagnosis of human disorders, particularly cancers (Wang et al., 2018). Although in some cases alterations in the levels of miRNAs in the peripheral blood might be solely a by-products of the underlying condition, many of these miRNAs have been shown to participate in the occurrence and development of cancer through direct or indirect routes. They can be used as tools for subtype classification of tumors, detection of resistance to chemo- or radiotherapy and clinical outcome (Wang et al., 2018). However, there is an urgent need for conduction of large scale studies to enhance the sensitivity, specificity, and applicability of these markers.

miR-135 is an example of miRNAs with diverse roles in both neoplastic and non-neoplastic conditions and possible application as a maker for both kinds of disorders. This miRNA is encoded by three different loci, namely *MIR135A1* (3p21.2), *MIR135A2* (12q23.1) and *MIR135B* (1q32.1). While *MIR-135A1* and *MIR-135A2* are located at different chromosomes in humans, they are transcribed into miR-135a. *MIR-135B* is the only gene accountable for miR-135b expression in humans.

miR-135b has been found to be expressed in brain, cerebellum, artery, colon, lung, stomach, esophagus, thyroid, salivary gland, breast, ovary, prostate and testis. miR-135a has a broader range of tissue expression. In addition to these tissues, it is expressed in whole blood, spinal cord, tibial nerve, heart, skeletal muscle, small intestine, adipocyte, kidney, liver, lung, spleen and a number of other tissues (GeneCards, 2017).

In the current review, we summarize the role of miR-135 in the carcinogenesis as well as development of other disorders.

## miR-135 in cancers

### Digestive tract cancers

*In silico* analyses using the Cancer Genome Atlas (TCGA) data on gastrointestinal cancers (including those originated from colon, esophagus, liver, pancreas, rectum and stomach) has shown that over-expression of miR-135 in the cancerous tissues is associated with a poor overall survival in these types of cancers. These significant findings were based on the receiver operating characteristic curves and Kaplan-Meier analyses which included 1,488 patients with gastrointestinal cancers whose data on survival and miR-135 expression was available in the TCGA (Chao et al., 2019).

### Colon cancer

In a pioneer study in this field, Nagel et al. have shown that miR-135a and miR-135b target the 3′ UTR of APC to decrease expression of this tumor suppressor gene and enhance activity of Wnt pathway. Authors have also reported significant over-expression of miR-135a and miR-135b in colorectal adenomas and carcinomas in correlation with down-regulation of APC transcripts (Nagel et al., 2008). Subsequently, Valeri et al. have shown that APC loss induces up-regulation of miR-135b leading to dysregulation of PTEN/PI3K pathway and up-regulation of SRC which in turn promotes cell transformation and progression of colorectal cancer. Over-expression of miR-135b has been found to be a common finding in sporadic and inflammatory bowel disease-associated human colorectal cancers. Moreover, its over-expression is correlated with tumor stage and poor patients’ survival. Suppression of miR-135b in animal models of colorectal cancer could reduce tumor growth through modulation of genes participating in proliferation, invasion, and apoptosis (Valeri et al., 2014).

### Gastric cancer

Bai et al. have shown that the exosome-mediated delivery of miR-135b to gastric cancer cells enhances angiogenic processes in these cells both *in vitro* and *in vivo*. Tumor cells-derived miR-135b could suppress expression of FOXO1 transcription factor that regulates gluconeogenesis and glycogenolysis by insulin signaling. This miRNA can also increase development of blood vessels (Bai et al., 2019). Huangfu et al. have shown that up-regulation of miR-135b enhances cell proliferation, migratory aptitude and invasive properties of gastric cancer cells through targeting the tumor suppressor gene CAMK2D which is a serine/threonine protein kinase pertaining to the subfamily of Ca2+/calmodulin-dependent protein kinases. Notably, *in vivo* administration of miR-135b antagonist has suppressed tumor growth and metastatic potential of tumors in xenograft models (Huangfu et al., 2021). Another study in early gastric cancer has reported down-regulation of miR-135a in about one-third of patients. Notably, these patients have exhibited advanced TNM stage and higher possibility of lymph node metastasis compared with patients having high levels of miR-135a. Functional studies have revealed that miR-135a suppresses viability of cells, EMT, invasive properties, and their migration. The rho-associated, coiled-coil-containing protein kinase 1 ROCK1 has been identified as the target of miR-135a in gastric cancer cells (Shin et al., 2014a). Similar to this report, He et al. have demonstrated down-regulation of miR-135 in gastric cancer tissues compared with adjacent tissues. Down-regulation of this miRNA has been associated with lower overall survival rate of patients. The impact of miR-135 on reduction of proliferation, invasion and migration of gastric cancer cells has also been confirmed in BGC-823 and SGC-7901 cell lines. Moreover, it has been revealed that this miRNA has a regulatory role on expression of the GTP exchange factor SMAD2 (He et al., 2019). On the other hand, Han et al. have found that miR-135b is the mostly up-regulated miRNA in gastric tissues from K19-C2mE and Gan mice. Moreover, expression of this miRNA has been shown to be elevated during the early stages of gastritis-associated carcinogenesis. This miRNA has also been shown to be up-regulated in gastric tumor tissues from gp130 F/F mice and human clinical samples. Interleukin 1 could enhance expression of this miRNA in gastric organoids and immortalized cell lines. Oncogenic effects of miR-135b in gastric cancer have been exerted through targeting FOXN3 and RECK tumor suppressors (Han et al., 2019).

### Pancreatic cancer

Zhou et al. have shown up-regulation of miR-135b in pancreatic cancer tissues and pancreatic cancer stem cells (CSCs). This miRNA has been shown to target the apoptosis and differentiation gene JADE-1. Up-regulation of miR-135b has increased proliferation, migratory potential, and invasion of pancreatic CSCs, suppressed their apoptosis and surged levels of stemness-related factors. Furthermore, miR-135b has been shown to enhance phosphorylated levels of AKT and mTOR. *In vivo* studies have also confirmed the impact of miR-135b up-regulation on acceleration of tumor growth (Zhou et al., 2020). Contrary to this study, Zhang et al. have shown down-regulation of miR-135a in pancreatic cancer tissues and cell lines compared with the corresponding controls. Luciferase activity assay has shown the interaction between long non-coding RNA (lncRNA) UCA1 and miR-135a. Notably, miR-135a could reverse the impact of UCA1 on apoptosis and viability of pancreatic cancer cells (Zhang et al., 2017a).

### Gallbladder cancer

Zhou et al. have found that expression of miR-135a-5p is repeatedly diminished in gallbladder cancer tissues in correlation with histologic grade. Forced over-expression of miR-135a-5p has suppressed proliferation of gallbladder cancer cells *in vitro* and *in vivo*. Furthermore, the cell surface protein participating in receptor-mediated endocytosis VLDLR has been identified as a direct target of miR-135a-5p. The p38 MAPK pathway has been found to participate in miR-135a/VLDLR downstream signaling (Zhou et al., 2014). Consistently, miR-135a has been shown to inhibit invasiveness and metastasis of gallbladder cancer cells and induce their apoptosis through regulation of ROCK1, HOXA10 and BCL-2 expression levels. miR-135a-loaded liposomes adapted with Anti-EGFR antibodies could decrease tumor growth in xenograft models (Feng et al., 2020).

## Hormone-dependent cancers

### Breast cancer

**FIGURE 1:**
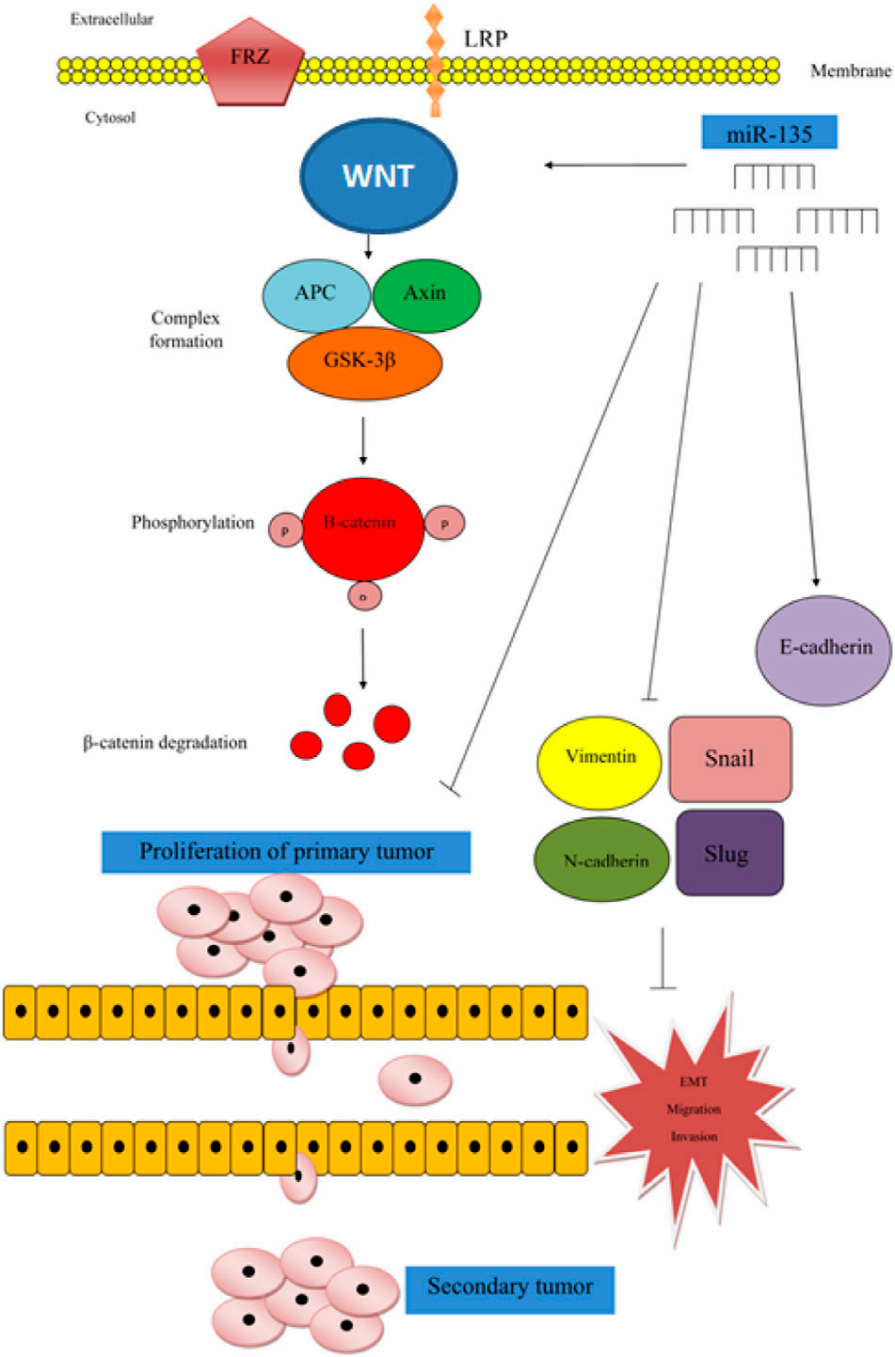
In breast cancer, miR-135 is regarded as a tumor suppressor miRNA, since over-expression of miR-135 could suppress cell proliferation, migration, invasion and EMT by inhibition of Wnt/β-catenin axis. In fact, expression of miR-135 has been positively associated with expression of p-GSK3, but inversely associated with expressions of Wnt and β-catenin. Up-regulation of miR-135 has increased p-GSK3 levels (Jiang et al., 2019). Mechanistically, GSK-3β participates in the formation of a complex with APC and Axin which has a role in β-catenin phosphorylation and its degradation (Wu and Pan, 2010).

Jiang et al. have reported a tumor suppressor role for miR-135 in human breast cancer cell lines as well as mice models. They have reported that forced over-expression of miR-135 suppresses growth, migratory aptitude, invasiveness and epithelial-mesenchymal transition (EMT) of MDA-MB-468 and MCF-7 cell lines. Mechanistically, miR-135 can suppress activity of Wnt/β-catenin pathway (Figure 1) (Jiang et al., 2019).

Similarly, Yang et al. have reported down-regulation of miR-135-5p in breast cancer samples compared to neighboring breast tissues with a more prominent reduction in patients having lymph node involvement. On the other hand, expression of SMAD3 has been shown to be increased in cancerous samples. Functional studies have shown that miR-135-5p inhibits TGF-β-mediated EMT, thus reducing metastatic ability of breast cancer cells *in vivo*. Besides, SMAD3 silencing has led to a similar phenotype with miR-135-5p up-regulation in breast cancer cells. Additional mechanistical assays have revealed that SMAD3, a fundamental modulator of TGF-β/SMAD signaling is directly targeted by miR-135-5p (Yang et al., 2020a).

In an attempt to find miRNAs regulating expression of the bone-metastasis promoting factor Runx2, Taipaleenmäki et al. have reported the inhibitory role of miR-135 on this factor in breast tissues. While miR-135 has been reported to be highly expressed in normal breast epithelial cells, it has not been expressed in metastatic breast cancer cells and clinical samples with high expression of Runx2. Forced over-expression of miR-135 in metastatic MDA-MB-231-luc cells has decreased expression of Runx2 and levels of pro-metastatic targets of Runx2, namely IL11, MMP-13, and PTHrP. These effects have been accompanied with reduction of tumor growth and bone metastasis in animal models (Taipaleenmäki et al., 2015).

Through a bioinformatics approach, Bertoli et al. have identified miR-135b as one of miRNAs with essential roles in regulation of the reformed functional pathways in basal type of breast cancer. With a degree centrality of 12, miR-135b has been shown to control expression of 12 over 35 genes inside 1 couple pathways (ethanol degradation X and Mismatch repair in eukaryotes). Experiments have confirmed over-expression of miR-135b in BT20 and MDA-MB-231 breast cancer cell lines compared to normal-like cell line of breast, i.e. MCF10A (Bertoli et al., 2021). Consistent with this study, Uva et al. have reported that expression levels of miR-135b are firmly correlated with triple negative breast cancer (TNBC) with basal-like phenotype. Target analyses of miR-135b have shown impact of this miRNA on TGF-β, WNT and ERBB pathways. Moreover, miR-135b expression has been correlated with neoplastic proliferative index (Uva et al., 2018).

Aakula et al. have shown that miR-135b has binding sites for 3′ UTR of androgen receptor (AR), estrogen receptor (ER) and HIF1AN and acts as a regulator of these genes. This miRNA has higher expressions in patients with ER negative breast tumors and in prostate cancer patients with low levels of AR (Aakula et al., 2015). In addition, Tribollet et al. have discovered a negative relationship between ER with miR-135 expression in breast and prostate cancers. miR-135a has an ability to suppress invasion and aggressiveness of these malignant cells (Tribollet et al., 2016).

## Genitourinary cancers

### Bladder cancer

miR-135 has been shown to be increased in bladder cancer cell lines and clinical samples. Up-regulation of miR-135a has enhanced proliferation of bladder cancer cells, while suppression of miR-135a has reversed this effect. PHLPP2 and FOXO1 have been identified as direct targets of miR-135a whose expressions are decreased by miR-135a. Cumulatively, miR-135a can promote proliferation in bladder cancer cells through decreasing levels of PHLPP2 and FOXO1 (Mao et al., 2015). Consistent with this study, Mao et al. have reported up-regulation of miR-135a, β-catenin, cyclin D1 and vimentin in bladder cancer samples compared with non-cancerous controls. Moreover, they have reported down-regulation of GSK3β and E-cadherin in cancerous samples. Functional studies have shown the role of miR-135a in acceleration of EMT, invasion and migratory potential of bladder cancer cells through enhancing activity of Wnt/β-catenin signaling. These effects are mediated via GSK3β down-regulation (Mao et al., 2018). Another study in bladder cancer cells has shown interaction between miR-135a and lncRNA MBNL1-AS1. In fact, the tumor suppressor role of MBNL1-AS1 is exerted through decreasing miR-135a levels and influencing activity of PHLPP2/FOXO1 axis (Wei et al., 2020).

## Other types of cancers

### Lung cancer

Tian et al. have shown down-regulation of miR-135a in non-small cell lung cancer (NSCLC) cells compared with normal bronchial epithelium. miR-135a has been found to inhibit proliferation, invasiveness and metastatic ability of NSCLC cells. Notably, miR-135a could suppress expression of several molecules in the RAS signaling pathway *via* suppression of expression of RAB1B (Tian et al., 2020). On the other hand, Zhao et al. have reported a pro-oncogenic role for miR-135b in NSCLC. They have also reported association between up-regulation of miR-135b and poor prognosis in these patients. The pro-proliferative, pro-angiogenic and anti-apoptotic effects of miR-135b have been verified in animal models as well. miR-135b has been shown to directly target the deubiquitinase CYLD transcript, thus controlling ubiquitination and activation of NF-κB pathway. Expression of this miRNA has been shown to be enhanced by IL-6/STAT3 axis. Cumulatively, IL-6/STAT3/miR-135b/NF-κB constitutes a positive feedback circuit which contributes in the development of NSCLC (Zhao et al., 2021).

Studies in prostate, renal and nasopharyngeal cancers as well as glioma have shown down-regulation of miR-135 in cancerous tissues compared with controls. These studies have also shown the impact of miR-135 down-regulation on enhancement of cell proliferation and aggressive behavior. On the other hand, miR-135 has been shown to be up-regulated in oral, cervical and liver cancers as well as myxoid liposarcoma, multiple myeloma and melanoma. Supplementary Table S1 summarizes the role of miR-135 in diverse types of cancers.

miR-135a has been shown to contribute to paclitaxel resistance in various tumor cells possibly through down-regulation of APC (Holleman et al., 2011). On the other hand, miR-135 has been reported to suppress tumor growth and cell invasion, and increase sensitivity to 5-fluorouracil and doxorubicin drugs through targeting FAK (Golubovskaya et al., 2014). Table 1 shows the effects of miR-135 in regulation of response to chemotherapy, radiotherapy and other therapeutic modalities.

**TABLE 1:** Effect of miR-135 in the response to chemotherapy, radiotherapy and other therapeutic modalities.

Type of cancer	Expression pattern*	Anticancer modality	Samples	Cell lines	Target	Other related molecules and pathways	Function	Ref
Prostate Cancer (PCa)	miR-135a-3p (-)	Tanshinone I	_	PC-3, M2182, DU145	DR5, caspase3, caspase8, cleaved PARP, BAX, Bcl-2, and Bcl-xL	TRAIL	Up-regulation of miR-135a-3p via Tanshinone I and TRAIL could enhance apoptosis via DR5 activation	Shin et al. (2014b)
Ovarian Cancer	miR135a-3p (down)	cisplatin and paclitaxel	Sera, peritoneal fluid, and tissue samples from 24 benign ovarian tumors, 7 benign gynecologic diseases, 157 ovarian cancers, and 5 normal ovaries/mice	SKOV-3, ES-2, OVCAR-3, RMG-1, 293T	PPP2R2B, BIRC3, GABRA3, and SPANXB1/2	_	Over-expression of miR-135a-3p induced cell sensitivity to cisplatin and paclitaxel and inhibited cell proliferation	Fukagawa et al. (2017)
Glioblastoma Multiforme (GBM)	miR-135b (up)	Radiotherapy	30 pairs of GBM tissues and ANCTs	U87, U87R	GSK3β	_	miR-135b by GSK3b targeting could promote cell radioresistance	Xiao et al. (2014)
Gastric Cancer (GC)	miR-135b-5p (up)	cisplatin	27 GC samples, 54 normal stomach samples/mice	MKN45, MKN28, SNU1, SNU601, AGS, STKM2, WD49074, WD50964, WD51064, WD51700	KLF4, and cleaved PARP	NF-kB-p65 pathway, and TNFα	H.*pylori* infection or TNFα could induce miR-135b-5p via NF-kB-p65 pathway activation and promote cell resistance to cisplatin	Shao et al. (2019)
	miR-135a (up)	oxaliplatin	280 pairs of GC tissues and ANCTs/mice	SNU-5, NCI-N87, MGC-803, SGC7901	E2F1, Sp1, DAPK2, p-gp, VEGF, cleaved PARP, and caspase3	c-MYC	miR-135a up-regulation via c-MYC could decline sensitivity to oxaliplatin and promote cell proliferation and suppress apoptosis by E2F1 and DAPK2/SP1 inhibition and also P-gp enhancement	Yan et al. (2016)
Esophageal Cancer (EC)	miR-135b-5p (up)	cisplatin	160 EC tissues, 11 normal tissues/mice	Eca109, KYSE150, EC9706, Het-1A, HEK-293T	TXNIP, ki67, Bax, and Bcl2	_	Cisplatin via miR-135-5p suppression and TXNIP enhancement could act against tumorigenesis	Di et al. (2021)
Non-Small Cell Lung Cancer	miR-135a (-)	gefitinib	_	NCI-H1650, NCl-H1975	RAC1, and B7-H4	PI3K/AKT	miR-135a via up-regulation of RAC1 and activation of PI3K/AKT could cause cell growth, viability, migration, invasion, metastasis and resistance to gefitinib	Zhang and Wang, (2018)
	miR-135 (up)	gefitinib	_	WI-38, A549, H1650, H1975, H157, H4006	TRIM16, E-cadherin, β-catenin, PD-L1, Bcl-2, Bax, caspase 9, caspase 3, p-JAK1, p-STAT1, and p-STAT2	JAK/STAT	miR-135 inhibition could restrain cell viability, migration, and invasion, while promote cell death and sensitivity to gefitinib via TRIM16 and JAK/STAT targeting	Wang and Zhang, (2018)
	miR-135b (up)	Radiotherapy	31 pairs of NSCLC tissues and ANCTs/mice	A549, H1975, 16HBE	_	LncRNA GAS5	GAS5 via miR-135b downregulation could suppress tumor progression, and increase the radiosensitivity of malignant cells	Xue et al. (2017)
Pancreatic Ductal Adenocarcinoma (PDAC)	miR -135b-5p (down)	Sulforaphane	6 PDAC tissues, 6 IPMN tissues, 6 normal tissues/chicken egg	AsPC-1, BxPC-3, PANC-1, MIA-PaCa2, BX-Gem	RASAL2, vimentin, p-ERK, ki67, CD133, CD44, cleaved caspase3, and E-cadherin	ERK1/2	miR-135b-5p enhancement by Sulforaphane could increase RASAL2 and hamper ERK1/2 pathway and tumor growth	Yin et al. (2019a)
Colon cancer	miR‐135b (up)	SD‐208	Mice	SW‐48	APC, FOXO1, RUNX1, and ESRRA	TGF‐β signaling	SD‐208 via TGF‐β pathway targeting could down‐regulate expression of miR‐135b and inhibit tumorigenesis	Akbari et al. (2015)
	miR‐135a (-)	Butyrate and trichostatin A (TSA)	_	LT97, HT29	P21	_	Butyrate and trichostatin A (TSA) reduced the expression of miR-135. miR-135a over-expression decreased butyrate and TSA-mediated inhibition of cell proliferation	Schlörmann et al. (2015)
	miR -135b (up)	doxorubicin	18 pairs of CRC tissues and ANCTs/mice	SW480, LOVO, COLO205, HT29, HEK-293	LATS2, and cleaved caspase-3	_	miR-135b through LATS2 targeting could promote the proliferation of cells and doxorubicin resistance while restrain apoptosis	He et al. (2015)

*Expression of this miRNA, in tumoral tissues has been compared with its expression in normal/non-cancerous tissues of the same origin.

## ANCT: Adjacent non-cancerous tissue, IPMN: Intraductal papillary mucinous neoplasm

### Clinical significance of miR-135

Several studies have verified correlations between serum/tissue levels of miR-135 and patients’ survival (Table 2). In a cohort of breast cancer patients, a positive correlation has been detected between miR-135b expression and ki67 expression, and a negative correlation has been reported between miR-135b expression and age and androgen receptor expression (Ghafouri-Fard et al., 2021b). Other studies have also reported association between expression of this miRNA and tumor size, tumor grade or other clinical data (Table 2).

**TABLE 2:** Prognostic role of miR-135 in the diseases.

Samples	Kaplan meier	Association with clinical data	Ref
63 BLBC tissues, 43 QNBC tissues, and 9 normal breast tissues	_	There was a positive association between miR-135b expression with ki67 expression, and negative association between miR-135b expression with age and androgen receptor expression	Ghafouri-Fard et al. (2021b)
32 primary PCa and 14 nonmalignant tissues		miR-135b level was associated with stage and the level of prostate-specific antigen (PSA)	Chao et al. (2019)
23 pairs of gallbladder cancer tissues and ANCTs	_	miR-135b level was associated with tumor grade	Bai et al. (2019)
33 pairs of glioma tissues and ANCTs	_	miR-135b level was associated with grade and tumor size	He et al. (2019)
59 pairs of early gastric cancer tissues and ANCTs	_	There was a negative correlation between miR-135b expression with lymph node metastasis and stage	Zhou et al. (2020)
146 gastric cancer (GC) tissues	The level of miR-135b was closely associated with prognosis	There was a strong correlation between miR-135b expression with stage, lymph-node metastasis, and local invasion	Zhou et al. (2014)
40 pairs of GC tissues and ANCTs	Low level of mir-135a was associated with poor prognosis	There was a correlation between low miR-135b expression and tumor metastasis	Jiang et al. (2019)
176 pairs of GC tissues and ANCTs	Low level of miR-135a was associated with poor survival	Low level of miR-135a was associated with TNM stage	Taipaleenmäki et al. (2015)
112 pairs of osteosarcoma tumors and ANCTs	High level of mir-135a was associated with lower overall survival and recurrence free survival	_	Mao et al. (2015)
19 myxoid liposarcoma tissues	High expression of miR-135b was correlated with poor survival	_	Mao et al. (2018)
98 pairs of non- small cell lung cancer (NSCLC) tissues and ANCTs	Low level of miR-135a was associated with poor overall survival and relapse-free survival	_	Li et al. (2015)
128 pairs of NSCLC tissues and ANCTs	High level of miR-135b was associated with poor prognosis of the patients	_	Chen et al. (2012)
67 cutaneous squamous cell carcinoma (cSCC) tissues	_	miR-135b expression inversely was correlated with tumor grade	Wang et al. (2016)
120 pairs of HCC tissues and ANCTs	_	There was a reverse correlation between miR-135b expression with HBeAg and capsule occurrence	Magalhães et al. (2018)
103 pairs of hepatocellular carcinoma (HCC) tissues and ANCTs	The high expression of miR-135b was correlated with poor survival	There was a correlation between miR-135b expression and stage, microvascular invasion, tumor recurrence, AFP level, HBs-Ag, hepatitis virus status	Xie et al. (2019)
65 pairs of lung adenocarcinoma (LUAD) tissues and ANCTs	_	High level of miR-135b was associated with advanced age	Zhao et al. (2019a)
Serum from 98 ovarian cancer patients	High expression of miR-135a-3p was associated with good prognosis	_	Hu et al. (2014)
280 pairs of GC tissues and ANCTs	High level of miR-135a in GC was associated with short survival and more recurrence probability	High miR-135a expression was associated with high level of CEA, vascular invasion, lymphatic metastasis, and weak differentiation	Lee and Park, (2017)
6 pancreatic ductal adenocarcinoma (PDAC) tissues, 6 IPMN tissues, and 6 normal tissues	_	Absent or low miR-135a expression was associated with high tumor grade	Petracco et al. (2019)
18 pairs of colorectal cancer (CRC) tissues and ANCTs	High miR-135a expression was associated with lower patients’ life expectancy	High miR-135a expression was associated with advanced stage (III)	Wang et al. (2021)
Digestive cancer system patients	High level of miR-135 expression was associated with poor OS, DFS, and RFS.	_	Chao et al. (2019)
Blood sample from 117 colon cancer patients and 120 normal subjects	High level of miR-135a was associated with good prognosis	miR-135a was related to stage, tumor type, tissue type, lymphatic metastasis, invasion, and differentiation degree	Zhou et al. (2021)
52 pairs of CRC tissues and ANCTs	_	miR-135b level was positively correlated with the stage, liver metastasis, and degree of malignancy	Xu et al. (2012)
Serum sample from 108 multiple myeloma (MM) patients and 44 healthy donors	_	High level of miR-135b was highly correlated with the severity of bone lesions	Hao et al. (2016)

## ANCT: Adjacent non-cancerous tissue, OS: Overall survival, DFS: Disease-free survival, RFS: Recurrence-free survival, BLBC: Basal-like breast cancer, QNBC: Quintuple negative breast cancer, IPMN: Intraductal papillary mucinous neoplasm

### miR-135 in non-cancerous conditions

miR-135 has diverse roles in the pathogenesis of human disorders. miR-135b acts as a neuroprotective miRNA via targeting GSK3β, thus it could neutralize effect of MPP + on proliferation and apoptosis of cells (Zhang et al., 2017b). Moreover, experiments in Zebrafish model have shown that miR-135a shields neural crest cells against alcohol-induced apoptosis and craniofacial deformities through regulation of Siah1/p38/p53 axis (Yuan et al., 2020).

**FIGURE 2:**
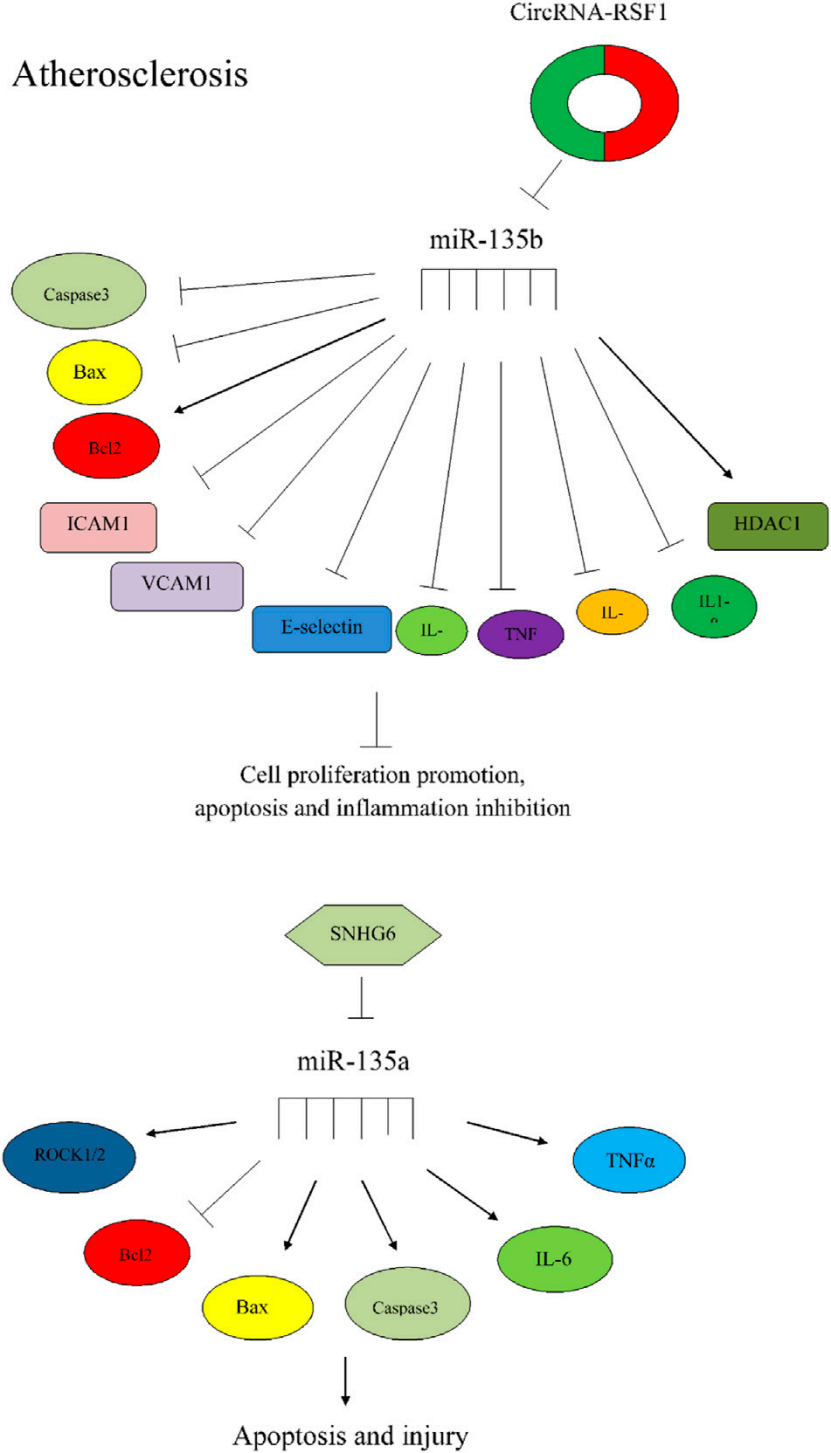
The importance of miR-135 in atherosclerosis. Circular RNA-RSF1 could promote proliferation of vascular endothelial cells and prevent apoptosis and inflammation via regulation of miR-135b-5p, HDAC1, ICAM1, VCAM1, caspase3, bcl2, and Bax (Hu et al., 2019). SNHG6 could increase apoptosis and injury via miR-135a-5p sponging and regulation of expression of ROCK1/2 and apoptotic factors (Ren et al., 2015).

miR-135 can also affect the process of atherosclerosis through different mecahnisms (Figure 2).

Table 3 shows the role of miR-135 in non-cancerous disorders.

**TABLE 3:** miR-135 in non-cancerous diseases.

Type of disease	Type/expression pattern*	Samples	Cell lines	Targets	Pathways	Function	Ref
Alzheimer (AD)	miR-135a-5p (down)	4 frontal cortex from AD patients and 4 controls/mice	N2a, and HEK293T	Rock2, p-Add1, Gsk3b, Bace1, Grin2b, Vldlr, Gga1, Fermt2, Ndufb9, Ndufa4	Foxd3, and tau	Inhibition of miR-135a-5p by Foxd3 could induce Rock2 expression and also phosphorylation of Add1 that lead to memory impairment and synaptic disorder	Zheng et al. (2021)
Atherosclerosis	miR-135a-5p (down)	Peripheral blood samples from 32 atherosclerosis patients, and 20 healthy donors	HUVEC and HEK 293T	ROCK1/2, IL-6, TNF-α, caspase-3, Bax, Bcl-2	SNHG6	SNHG6 could aggravate injury via miR-135a-5p sponging to up-regulate ROCK1/2 expression	Shan et al. (2020)
	miR-135b-5p (-)	**_**	HUVEC	HDAC1, Bax, cleaved caspase-3, VCAM1, ICAM1, E-selectin, Bcl-2, IL-1β, IL-6, TNF-α, IL-8	CircRNA RSF1	CircRSF1 could promote vascular endothelial cell proliferation and prevent apoptosis and inflammation via miR-135b-5p/HDAC1 regulation	Zhang et al. (2021)
	miR-135b-5p (up)	Serum sample from 90 atherosclerotic patients, 50 healthy volunteers, and 15 non-atherogenic cardiovascular patients	HEK293, HUVEC, and VSMC	MEF2C, FOXN3	_	miR-135b-5p could promote cell proliferation and migration by repressing MEF2C	Xu et al. (2015b)
	miR-135a (down)	_	Rat VSMCs	KLF4, STAT3, ALP, OC	_	miR-135a by KLF4/STAT3 targeting could decrease cell calcification	Lin et al. (2016)
Depression	miR-135a (down)	blood sample from 50 patients with depression, and 50 healthy volunteers/mice	_	TLR4, IL-1β, IL-6, TNF-α, Bax, and Bcl-2	_	miR-135a over-expression could prevent cell apoptosis, and reduce the expression levels of inflammatory factors and TLR4	Ding et al. (2021)
	miR-135a (down)	Blood samples from 39 cases and 36 normal controls	_	_	_	miR-135a could be a potential biomarker of depression	Gheysarzadeh et al. (2018)
Long-term Depression (LTD)	miR-135 (up)	Mice, rat	COS-7, primary hippocampal neurons	complexin-1/2	_	miR-135 over-expression via NMDA treatment could decrease complexin-1/2 and required for maintenance spine restructuring in LTD.	Hu et al. (2014)
Diabetes	miR-135a (down)	Mice	HL-1	TXNIP	_	miR-135a overexpression could guard myocardial cells from mI/R injury by TXNIP targeting	Liu et al. (2015)
Parkinson	miR-135b-5p (-)	_	SK-N-SH and SK-N-BE	GPNMB, PCNA, Bax, cleaved caspase3, Bcl2	LncRNA MALAT1	MALAT1 downregulation could promote cell proliferation while prevent apoptosis by miR-135b-5p/GPNMB axis regulation	Lv et al. (2021)
Pulmonary Arterial Hypertension (PAH)	miR-135a (up)	Mice	_	BMPR2	_	miR-135a downregulation could decrease the PAH phenotype via recovering of BMPR2	Lee and Park, (2017)
	miR-135a-5p (up)	Rat	pulmonary artery smooth muscle cell (PASMC)	TPRC1	_	Up-regulation of miR-135a-5p by hypoxia or monocrotaline (MCT) could cause TPRC1 and proliferation inhibition	Liu et al. (2019)
Pulmonary Fibrosis	miR-135a (down)	Mice	BEAS-2B, and HEK293T	TLR4, α-SMA, E-cadherin, p-p65, IL-1β, TNF-α, TGF-β, p-IKKα, p-IKBα, IFN-γ	NF-κB	miR-135a could inhibit NF-κB by targeting TLR4 for improvement of inflammatory response and pulmonary fibrosis	Xie et al. (2020)
Osteonecrosis of Femoral Head (ONFH)	miR-135b (down)	Rat	MG-63 and U-2	PDCD4, caspase-3, OCN	_	HiPS-MSC-Exos and miR-135b could promote cell proliferation and restrain apoptosis via inhibition of PDCD4, as a result reduce the bone loss	Zhang et al. (2020b)
Nephrotic Syndrome (NS)	miR-135a -5p (down)	Plasma from 52 NS cases and 24 healthy controls	_	GSK-3β	_	Dysregulated expression of miR-135a-5p and its target GSK-3β could have a role in the pathogenesis of NS.	Ardalan et al. (2020)
Endometriosis	miR-135a/b (down)	23 pairs of endometriosis lesions and ANCTs	_	HOXA-10	_	Expression levels of miR-135a/b in endometriosis lesions were decreased but were increased in the secretory phase compared with the proliferative phase in endometriosis lesions	Petracco et al. (2019)
	miR-135a/b (up)	Endometriosis lesions from 32 patients and 50 endometrial biopsies from healthy controls	MCF-7, Primary endometrial stromal cell	HOXA10	_	miR-135a/b inhibition could cause HOXA10 upregulation and help for treatment of endometriosis-associated infertility	Petracco et al. (2011)
	miR-135a (up in eutopic and down in ectopic)	Tissues from 17 endometriosis cases and 17 normal endometrials from healthy controls	_	HOXA10	_	miR-135a may be considered as an endometrial biomarker in the in the early stages of endometriosis	Mirabutalebi et al. (2018)
Epilepsy	miR-135a-5p (up)	_	BV2	SIRT1, caspase-3, caspase-9	_	miR-135a-5p inhibition could increase proliferation and protect nerve cells against apoptosis	Wang et al. (2021)
Allergic Rhinitis (AR)	miR-135a (down)	Mice	_	GATA-3, T-bet	_	miR-135a through GATA3 targeting and equilibration in Th1/Th2 could regulate the immune system and suppress inflammation	Deng et al. (2015)
Non-alcoholic Fatty Liver (NAFLD)	miR-135a-3p (down)	Serum from 50 patients with NAFLD and 50 healthy individuals/mice	Hepatic cells	_	PDGFR, and AMPK signaling pathway	miR-135a-3p may act as a potential sensitive, specific and non-invasive biomarker in NAFLD.	Jiang et al. (2021)
Cerebral Ischemia	miR -135b-5p (-)	_	HT22	GSK-3β, Caspase-3 HO-1, NQO1	Nrf2/ARE	miR-135b-5p over-expression could protect neurons via GSK-3β targeting and the Nrf2/ARE signaling axis activation	Duan et al. (2018)
Acute Ischemic Stroke (AIS)	miR-135b (up)	Blood samples from 76 patients and 60 healthy controls	PC12	TRPC6	_	miR-135b up-regulation significantly could decrease the expression of TRPC6	Yang et al. (2020b)
Hypertension	miR-135a (down)	_	HeLa	NR3C2	_	miR-135a via NR3C2 targeting could regulate blood pressure	Sõber et al. (2010)
Systemic Sclerosis (SSc)	miR-135b (down)	5 tissues and 15 serum samples from SSc patients and 5 tissues and 12 serum samples from healthy controls/mice	_	STAT6, collagen	TGF-β, and MeCP2	miR-135b by silencing of STAT6 could reduce IL-13-induced collagen expression	O'Reilly et al. (2016)
Impaired Angiogenic –Related Diseases	miR-135a-3p (up)	Plasma from 20 acute coronary syndromes patients and 40 normal individuals, plasma and skin samples from 10 nondiabetic and 13 diabetic persons/mice	HUVEC	HIP1	p38K axis, bFGF, VEGF, TSP1, TSP2, and HDAC class IIa	miR-135a-3p through VEGF-HIP1-p38K signaling axis targeting could act as a angiogenic and tissue repair inhibitor	Icli et al. (2019)
Elevated Progesterone in Assisted Reproductive Technology (ART) Treatment	miR-135a (up)	10 women with elevated progesterone and 10 women with normal progesterone	Ishikawa	HOXA10, EMX2, ITG-3	_	miR-135a up-regulation by high progesterone could inhibit HOXA10 and as a result EMX2 promotion and ITGβ3 inhibition happened and endometrial receptivity affected	Luo et al. (2021)
Axon Injury	miR-135 (-)	Mice	SH-SY5Y, HEK293, and N2A	KLF4	_	miR-135a/b via KLF4 targeting could promote CNS Axon Growth and Regeneration	van Battum et al. (2018)
Chronic Intermittent Hypoxia (CIH)	miR-135a (down)	Mice	mice aortic endothelial tissues, and HEK293	HIF-1α, PCNA, Bcl-2, cleaved caspase 3, Bax	LncRNA MEG3	MEG3 via miR-135a/HIF-1α regulation could suppress cell proliferation and promote apoptosis and injury of aortic endothelial cells	Ding et al. (2020)
Podocyte Injury	miR-135 (up)	3 FSGS patients and 3 patients with kidney rupture/mice	MPC5, and 293T	GSK3β, desmin, snail, nephrin, E-cadherin, WT1	Wnt/β-catenin	miR-135 could promote severe podocyte injury through activation of Wnt/β-catenin signaling and GSK3β inhibition	Yang et al. (2015)
	miR-135a (up)	3 patients with FSGS and 3 patients with kidney rupture/mice	MPC5, and 293T	TRPC1, desmin, snail, nephrin, E-cadherin, caspase-3, WT1	TGF-β	miR-135a could cause severe podocyte injury and apoptosis via TRPC1 targeting	Yang et al. (2017)
Cutaneous Wounds	miR-135a (-)	Rat	hAMSC, BJ, 293 T	LATS2, E-cadherin, N-cadherin, α-SMA	_	miR-135a through LATS2 targeting could cause cutaneous wounds improvement	Gao et al. (2020)
Osteoporosis (OP)	miR-135b-5p (up)	Bone fragments from 30 patients with OP and 30 patients with osteoarthritis	MC3T3-E1	RUNX2, OC, Osterix, ALP	_	miR-135-5p through RUNX2 targeting and osteogenic differentiation and osteoblast growth inhibition and apoptosis induction could involve in osteoporosis	Chen et al. (2020)
	miR-135-5p (-)	_	MC3T3-E1	HIF1AN, ALP, Runx2, OSX, OPN, OCN	_	miR-135-5p by HIF1AN targeting could induce osteogenic differentiation and calcification	Yin et al. (2019b)

*Expression of this miRNA, in affected tissues/cells has been compared with its expression in normal tissues/cells of the same origin.

## mI/R: Myocardial ischemia/reperfusion injury, FSGS: Focal segmental glomerulosclerosis

According to Kim et al. study in mice models and RBL2H3, B16F1, B16F10 cell lines, miR-135-5p could prevent allergic inflammation through targeting P62 (Shan et al., 2020). Sung et al. have performed a series of assays in Neuro-2a cells and mice model. Their investigation has revealed that ischemic preconditioning as an endogenous neuroprotective process promotes expression of ATP-binding cassette subfamily A member 1 (ABCA1), suppresses miR-135-5p expression, decreases the Bax/Bcl2 proportion and activates caspase-9 and caspase-3, thus protecting neural cells against mitochondria-dependent apoptosis and subsequent brain injuries (Sung et al., 2021). According to Xie et al. study, miR-135b-5p inhibition could protect cells separated from myocardial tissues of mice from apoptosis and reperfusion injury by activating JAK2/STAT3 signaling axis (Xie et al., 2017).

In an interesting study, the relationship between physical exercise and miR-135 expression in old mice was identified. In this survey, authors have reported miR-135a-5p down-regulation *via* exercise. The consequent cell cycle progression and proliferation of neural precursor cells has led to neurogenesis. Phosphatidylinositol signaling protein IP3 has been recognized as a target of this miRNA, so miR-135a-5p/IP3 axis has been suggested as a potential target for treatment of age-related brain injuries (Pons-Espinal et al., 2019).

According to Honardoost et al. study, miR-135 transfection to C2C12 cell line caused Insr gene down-regulation, glucose uptake reduction and development of insulin resistance phenotype (Honardoost et al., 2016).

## Causes and consequences of aberrant miR-135 expression

miR-135 is a miRNA which is involved in the pathoetiology of several neoplastic and non-neoplastic conditions. Both tumor suppressor and oncogenic roles have been reported for this miRNA. Two studies in animal models of spontaneous carcinogenesis has reported oncogenic roles for miR-135b in colorectal (Valeri et al., 2014) and gastric (He et al., 2019) cancers. Since these two studies have provided the strongest level of evidence, oncogenic function is supported for this miRNA.

Studies in prostate, renal, gallbladder and nasopharyngeal cancers as well as glioma have shown down-regulation of miR-135 in cancerous tissues compared with controls. Meanwhile, miR-135 has been shown to be up-regulated in bladder, oral, colorectal and liver cancers. Studies in breast, gastric, lung and pancreatic cancers as well as head and neck squamous cell carcinoma have reported dual roles for miR-135. These different effects of miR-135 cannot be explained either by tissue-dependent factor or by different genetic loci that encode this miRNA (*MIR135A* versus *MIR135B*). Other explanations for these observations are the presence of tissue-dependent elements, abundance of RNA binding proteins in each tissue, impact of alternative splicing/polyadenylation events on miR-135 targets and the amounts of cell type-specific factors that change secondary structure of target transcripts.

Studies aimed at identification of the impact of miR-135 on EMT process have shown extremely contradictory results. For instance, miR-135-5p as a tumor suppressor miRNA inhibits this process (Yang et al., 2020a). On the contrary, in bladder and gastric cancer, miR-135a and miR-135b have been found to increase EMT (Mao et al., 2018; Huangfu et al., 2021).

Circulatory levels of miR-135 can be used as prognostic markers in different types of cancers. For instance, high expression of miR-135a-3p in serum samples of patients with ovarian cancer has been associated with good prognosis (Hu et al., 2014). Moreover, over-expression of miR-135a in blood samples of patients with colon cancer has been associated with good prognosis 83)). On the other hand, high serum levels of miR-135b in patients with multiple myeloma have been correlated with the severity of bone lesions (Hao et al., 2016).

Amplification or deletion of any of three mentioned loci for miR-135, namely *MIR135A1* (3p21.2), *MIR135A2* (12q23.1) and *MIR135B* (1q32.1) might be associated with dysregulation of certain members of this family. For instance, frequent deletion of the *MIR135A1* locus has been found to be associated with poor prognosis in primary breast cancers. Mechanistically, deletion of this locus and subsequent down-regulation of miR-135a levels enhances progression of ERα+ breast cancers and their resistance to tamoxifen (Zhang et al., 2018).

Studies in different tissues and cell types have identified common pathways (e.g. AKT and WNT) and target genes (such as APC, FOXO1, FOXN1, RECK and some MMPs) for miR-135. Identifying these common functions would also provide insight into the impact of exosomes-delivered miR-135 on function of distal recipient cells/tissues.

miR-135 has been shown to interact with a number of lncRNAs, namely MBNL1-AS1, MALAT1, UCA1, MEG3, DANCR, SMAD5-AS1, NCK1-AS1 and RAET1K. In fact, these lncRNAs exert their impacts on cellular functions through sponging miR-135. In addition, miR-135 has been found to modulate activity of several signaling pathways such as Wnt/β-catenin, TGF-β/SMAD, ERBB, PI3K, p38 MAPK EGFR, FAK, NF-κB, Notch, IL-6/STAT3, AKT/mTOR and Hippo. This extensive mode of action shows complexity of functional network influenced by miR-135.

Differences in pri- and pre-miRNA sequences indicate that diverse phases might be rate-limiting for each precursor, enabling the fruition of extra regulatory mechanisms. Moreover, miR-135 might be subjected to distinctive modes of regulation through certain interactions with different RNA-binding proteins.

Dysregulation of miR-135 has also been noted in various non-neoplastic conditions such as Alzheimer’s disease, atherosclerosis, depression, diabetes, Parkinson, pulmonary arterial hypertension, nephrotic syndrome, endometriosis, epilepsy and allergic conditions.

## Conclusion

Regarding the different roles of miR-135 in different contexts, several issues should be addressed about the possible effects of tissue-dependent elements that affect expression of miR-135 in each tissue. Although manipulation of expression of miR-135 is a possible therapeutic option for cancer, it is not expected that miR-135-targeted therapies enter clinical settings in near future.
