# Supplementary material for: A review on the importance of miRNA-135 in human diseases
Source: Front Genet. 2022 Sep 6;13:973585. doi: 10.3389/fgene.2022.973585 (PMC9486161; doi:10.3389/fgene.2022.973585)
Supplement: Supplementary file 1 [file Table1.docx]

Table 1 summarizes the role of miR-135 in diverse types of cancers.

Table 1. miR-135 in cancers.

| **Ref** | **Function** | **Pathways** | **Targets** | **Cell lines** | **Samples** | **miRNA expression*** | **Type of cancer** |
| --- | --- | --- | --- | --- | --- | --- | --- |
| **Studies in mouse models of spontaneous carcinogenesis** | | | | | | | |
| (12) | miR-135b is an important downstream effector of oncogenic pathways. | PTEN/PI3K | TGFβR2, DAPK1, APC | mouse embryonic fibroblasts having mutations in specific oncogenes, human CRC cell lines | 454 sporadic and 31 IBD-associated CRCs/ Conditional deletion of Apc deletion by b-napthoflavone injection in AhCre^+^Apc^+/+^ mice | miR-135b (up) | Colorectal Cancer (CRC) |
| (16) | miR-135B enhances invasiveness and stem-cell properties of gastric cancer cells. | IL-1 | FOXN3, RECK | SNU-719, SNU-601, SNU-638, and AGS | Murine gastritis (K19-C2mE) and gastric tumor (K19-Wnt1/C2mE; Gan) | miR-135b (up) | Gastric Cancer |
| **Studies in other animal models/clinical samples** | | | | | | | |
| (35) | CircNOL10 via miR-135a/b-5p/ KLF9 axis regulation could inhibit CRC progression. | CircNOL10 | KLF9, cyclinD1, c-myc, MMP9, and E-cadherin | SW620, SW480, LOVO, HCT116, NCM460 | 55 pairs of CRC tissues and ANCTs/mice | miR-135a/b-5p (up) | Colorectal cancer |
| (36) | miR-135b via TGFBR2 targeting could induce the cell proliferation and inhibit the apoptosis. | TGF-β signaling pathway | TGFBR2 | HT-29, SW-480 | 5 pairs of CRC tissues and ANCTs | miR-135b (up) |  |
| (24) | Overexpression of miR-135-5p could suppress EMT and metastasis by inhibition of SMAD3 and TGF-β/SMAD signaling axis. | TGF-β/SMAD | SMAD3, Snail, Vimentin | MCF-10A, MDA-MB-231, MCF-7, and 293T | 66 pairs of BC and ANCTs/ mice | miR-135-5p (down) | Breast cancer |
| (25) | miR-135 via BMP-Runx2 axis targeting could block proliferation, migration, growth and metastasis of cells. | BMP-Runx2 | Runx2, BMPIR, BMPIIR, SMAD4, SMAD5, SMAD1, SMAD8, ROCK1, CD44, PTK2, CCL7, CXCL12, IL-11, MMP-13, PTHrP, Id2 | MCF-10A, MCF-7, and MDA-MB-231-a/b | 4 pairs of primary tumors and bone metastasis, 2 normal bones /mice | miR-135 (down) |  |
| (26) | miR-135b inhibition could suppress cell proliferation, migration, invasion, and MET. | Ethanol degradation, and mismatch repair | ACSL1/3, ALDH1A1/1B1/3A24A1, FEN1, MSH6, RFC2, RFC4, RPA1, SLC19A1, E-Cadherin | MDA-MB-231, BT20, MCF10A | 8 pairs of TNBC tissues and ANCTs/ mice | miR-135b (up) |  |
| (22) | Over-expression of miR-135 could suppress cell growth, migration, invasion and EMT by inhibition of wnt/β-catenin axis. | Wnt/β-catenin | p‑GSK3, MMP2, MMP9, E-cadherin Snail, Slug, N-cadherin, Vimentin | MDA-MB-468, MDA-MB-231, MCF-10A, MCF‑7 | Mice | miR-135 (down) |  |
| (27) | miR-135b could have an important role in breast cancer pathogenesis. | TGF-beta, WNT, and ERBB pathway | THBS1-2, TGFBR1-2, SMAD2-4, SP1, MYC, ROCK1-2, PP2A-P70S6K,  GSK-3β, CK1α, APC, SFRP4, SIAH1, CBL-b | _ | 63 BLBC tissues, 43 QNBC tissues, and 9 normal breast tissues | miR-135b (up in BLBC compared with others) |  |
| (37) | miR-135a by HOXA10 targeting could promote cell migration and invasion. | _ | HOXA10 | HEK293, BT549, SKBr3, MDA-MB-231, MCF7, T47d, HUVEC, and HBL-100 | 10 adenosis or fibroadenoma tissues, and 30 breast cancer tissues | miR-135a (up) |  |
| (31) | miR-135a over-expression could promote growth, proliferation, EMT, invasion and migration of cells, while prevent apoptosis. | Wnt/β-catenin | GSK3β, β-catenin, cyclinD1, vimentin, and E-cadherin | EJ, T24, BIU87, SCaBER, 5637, SVHUC-1 | 165 pairs of tumor tissues and ANCTs/mice | miR-135a (up) | Bladder Cancer |
| (32) | MBNL1-AS1 via miR-135a-5p/ PHLPP2/ FOXO1 axis could involve in BC pathogenesis. | LncRNA MBNL1-AS1, and AKT signaling pathway | PHLPP2, FOXO1, p-AKT, Ki67, cleaved caspase3, cleaved caspase9, Cyclin D1, p21, and p27 | 5637, T24, 293T | 21 pairs of tumor tissues and ANCTs/mice | miR-135a-5p |  |
| (30) | miR-135a by PHLPP2, and FOXO1 suppression could promote cell proliferation. | PI3K/AKT | PHLPP2, FOXO1, AKT, P21, P27, Ki67, and Cyclin D1 | EJ, T24, BIU87, SCaBER, and 5637 | 7 pairs of tumor tissues and ANCTs | miR-135a (up) |  |
| (38) | Over-expression of miR-135b via STAT6 targeting could prevent invasion and migration of malignant cells. | _ | STAT6, and  p-STAT6 | DU145,  PC3 | 32 primary PCa and 14 nonmalignant tissues | miR-135b (down) | Prostate Cancer (PCa) |
| (39) | miR-135b could involve in oral cancer development. | _ | APC | _ | 28 oral cancer tissues,  11 ANCTs, and 19 normal gingival tissues | miR-135b (up) | Oral Cancer |
| (40) | Over-expression of miR-135a could inhibit proliferation, invasion and induce cell arrest. | 25 various pathways | c-MYC, and CCND1 | caki-2, A498 | 38 pairs of RCC tissues and ANCTs | miR-135a (down) | Renal Cell Carcinoma (RCC) |
| (20) | Over-expression of miR-135a-5p could act against tumor by inhibition of cell proliferation. | p38 MAPK pathway | VLDLR, and SP3 | HEK293T, GBC-SD, EH-GB1, SGC-996 | 23 pairs of GBC tissues and ANCTs/ mice | miR-135a-5p (down) | Gallbladder Cancer (GBC) |
| (41) | miR-135a could hamper invasion and metastasis and increase apoptosis. | _ | ROCK1, HOXA10, BCL-2 | GBC-SD | GBC tissues and ANCTs/mice | miR-135a (down) |  |
| (21) | Over-expression of miR-135a-5p by ANGPT2 targeting could suppress cell proliferation, migration and invasion. | _ | ANGPT2, Rock1, and E-cadherin | GBC‑SD | 10 pairs of GBC tissues and ANCTs | miR‑135a‑5p (down) |  |
| (42) | DANCR knockdown via miR135a-5p/BMI1 pathway regulation could suppress cell proliferation, migration and invasion. | LncRNA DANCR | BMI1 | LN229, U251, NHAs | 33 pairs of glioma tissues and ANCTs/mice | miR-135a-5p (down) | Glioma |
| (43) | miR-135a could prevent proliferation and migration of cells via NHE9 and EGFR inhibition. | EGFR signaling | NHE9 | U87, U251, HEK293 | Brain tissue from normal individuals | miR-135a (down) | Glioblastoma |
| (15) | miR-135a via ROCK1 targeting could hamper cell viability, EMT, cell invasion, migration, and lymph node metastasis. | _ | ROCK1, E-cadherin, N-cadherin, and Slug | HFE145, AGS, YCC2, MKN28, KATOIII, SNU1, SNU5, SNU16, SNU216, SNU601, SNU638, SNU668, SNU719 | 59 pairs of EGC tissues and ANCTs | miR-135a (down in 33.9% and up in 66% of the patients with early gastric cancer) | Gastric cancer |
| (13) | miR-135b via FOXO1 inhibition and IL8 augmentation could cause tumor angiogenesis and cell growth. | _ | FOXO1, and IL8 | SGC7901, GES-1, HEK293T, HUVEC | 16 pairs of GC tissues and ANCTs, serum sample from 150 GC patients and 150 normal individuals /mice | miR-135b (up) |  |
| (14) | Overexpression of miR-135b could augment cell proliferation, EMT, migration and invasion. | _ | CAMK2D, vimentin, E-cadherin, and N-cadherin | MKN28 BGC823, GES-1, HEK293T | 28 pairs of GC tissues and ANCTs, Blood samples from 23 GC patients and 27 healthy controls/mice | miR-135b (up) |  |
| (44) | miR-135a could prevent tumor growth, migration, invasion and angiogenesis by FAK targeting. | FAK pathway, ROCK/LIMK pathway, and p53 | FAK, p-ERK, and VEGFA | MGC-803, BGC-823, SGC- 7901, MKN1, MKN45, GES-1, MDA-MB-435, HUVEC | 176 pairs of GC tissues and ANCTs/ mice | miR-135a (down) |  |
| (45) | Up-regulation of miR-135b could cause inhibition of apoptosis and promote migration, invasion and proliferation of cells. | β-catenin/Wnt | APC | AGP01, ACP02, ACP03 | _ | miR-135b-5p (up) |  |
| (46) | miR-135a could restrain migration and metastasis of cells via TRAF5-mediated NF-κB axis suppression. | NF-κB | TRAF5, ikβ, p-p65 MMP2, MMP9, ICAM-1, and VCAM-1 | AGS, BGC-823, MKN-28, MKN-45, SGC-7901, and GES-1 | 40 pairs of GC tissues and ANCTs | mir-135a (down) |  |
| (47) | miR-135 expression has a positive correlation with CEA and CA199 GC markers and could be used in GC diagnosis. | CEA, and CA199 | _ | _ | Blood samples from 78 GC patients, and 80 normal individuals | miR-135 (up) |  |
| (48) | miR-135b expression was positively associated with helicobacter pylori infection and may be involved in the immune response modulation in association with H.pylori infection. | _ | APC, and KLF4 | _ | 20 tissues of normal gastric mucosa, 20 non­atrophic chronic gastritis, 10 of intestinal metaplasia, and 14 gastric intestinal adenocarcinoma | miR-135b  (up in gastritis and metaplasia and down  in normal and  adenocarcinoma) |  |
| (49) | miR-135a-5p could restrain cell growth and promote apoptosis by targeting HOXA10. | _ | HOXA10 | CAL-27, NEC, Het-1A, FaDu, HEK293 | 43 pairs of HNSCC tumors and ANCTs/mice | miR-135a-5p (down) | Head and Neck Squamous Cell Carcinoma (HNSCC) |
| (50) | TGFBR1 deletion and as a result miR-135b overexpression could promote malignancy via FIH decline and HIF-1α enhancement. | TGFBR1 | FIH, HIF-1α, CBP/p300, and VEGFA | CAL27, SSC4, SCC9, SCC15, SCC25, HSC3, KCCT873, KCCOR891,OSC19, HOK, HUVEC | Mice | miR-135b (up) |  |
| (51) | ZFAS1 via regulation of miR-135a/ APEX1 axis could promote proliferation, migration and invasion and inhibit apoptosis. | LncRNA ZFAS1 | APEX1, Ki-67, CyclinD1, Bax, Bcl-2, MMP2, and MMP9 | MG63, hFOB1.19, 143B, U2OS, Saos2 | 34 tumors, 30 normal bone tissues from patients with simple fractures /mice | miR-135a (down) | Osteosarcoma |
| (52) | Over-expression of miR-135a by BMI1 and KLF4 targeting could inhibit growth, invasion, and lung metastasis. | _ | BMI1, KLF4, ki67, MMP2, and MMP9 | MG63, Saos2, and osteoblast-like cells | 10 tumors and 3 normal cartilages/mice | miR-135a (down) |  |
| (53) | miR-135b via GSK3β, CK1α, and TET3 inhibition could promote recurrence, stemness and metastasis. | Wnt/β-catenin, and Notch pathways | GSK3β, APC,  β-TrCP, CK1α, HES1, TET3, Nanog, BMI1, and Oct4 | MG63, hFOB, MNNG/HOS, Saos2, LM5, HuO9, M132 | 112 pairs of tumors and ANCTs /mice | miR-135b (up) |  |
| (54) | miR-135b via THBS2 suppression and MMP2 enhancement could promote lung metastasis and invasion of cells. | _ | THBS2, IL1R, and MMP2 | Human adipose-derived stem cell line, 2645-94, 1955-91, 402-91 | 27 FFPE samples from MLS patients, 30 MLS tumors, 25 adjacent normal muscle tissues/mice | miR-135b (up) | Myxoid Liposarcoma (MLS) |
| (55) | miR-135a over-expression via downregulation of FOXO1 could promote tumorigenicity. | PI3K/AKT | FOXO1, Cyclin D1, P21, and P27 | Human epidermal melanocytes (HEM), sk-mel-1,  A375, 293FT, HEK293 | 20 pairs of melanoma tissues and ANCTs | miR-135a (up) | Melanoma |
| (56) | miR-135a up-regulation via suppression of NCK1-AS1 could inhibit cell migration and invasion. | LncRNA NCK1-AS1 | _ | C666-1, 13-9B | Plasma from 50 NPC patients, 50 TMJ patients, and 50 healthy volunteers | miR-135a (down) | Nasopharyngeal Carcinoma (NPC) |
| (57) | DANCR via miR-135a-5p/ KLF8 axis could promote the malignancy. | LncRNA DANCR | KLF8, MMP-2, and MMP-9 | SCC9, TSCCA, TCa-8113, CAL-27, 293T | Mice | miR-135a-5p  (-) | Tongue Squamous  Cell Carcinoma (TSCC) |
| (58) | SMAD5-AS1 via miR-135b-5p/APC could prevent cell proliferation. | LncRNA SMAD5-AS1, and Wnt/β-catenin pathway | APC | TMD8, U2932, GM12878, HEK-293, OCI-Ly3, WSU-FSCCL, JeKo-1, L428, Raji, HEK-293FT | 11 pairs of DLBCL lymph glands and ANCTs/mice | miR-135b-5p  (-) | Diffuse B cell  Lymphoma (DLBCL) |
| (34) | miR-135b elevation by IL-6/STAT3 could promote proliferation, migration, invasion, and angiogenesis and inhibit apoptosis. | IL-6/STAT3, and NF-κB pathway | CYLD, Bcl-2, Bcl-xL, MMP9, A20, NFKBIA, VEGFC, IL-1β, IL-6, IL-8, cyclin D1, cleaved caspase3, ki67,RIP1, CD31, cleaved PARP, p-P65, and p-IKK | H1299, H1975, H292, H358, H460, H520, A549, PC-9, Calu-3, HUVEC, 293FT, BEAS-2B | 128 pairs of NSCLC tissues and ANCTs/ mice | miR-135b (up) | Non-Small Cell Lung Cancer (NSCLC) |
| (33) | miR-135a via RAB1B targeting could block proliferation, invasion and metastasis of the cells. | RAS pathway | RAB1B, RAS, Raf1, Rac1, and RhoA | A549, H1299, H1650, LTEP-a-2, HBE, HEK-293T | 98 pairs of NSCLC tissues and ANCTs | miR-135a (down) |  |
| (59) | RAET1K through miR-135a-5p sponging could regulate CCNE1 and stop cell cycle progression. | LncRNA RAET1K | CCNE1 | A549, H1299, PC-9 | 505 LUAD tissues, and 59 ANCTs | miR-135a-5p  (down) | Lung Adenocarcinoma (LUAD) |
| (60) | LUADs with EGFR mutations and miR‑135b overexpression are more likely for visceral pleura invasion. | EGFR signaling pathway | _ | _ | 65 pairs of LUAD tissues and ANCTs | miR-135b (up) |  |
| (61) | Suppression of miR-135b could result in up-regulation of LZTS1 and decrease cell motility and invasion. | _ | LZTS1 | MET1, MET4, PM1 | Tissue from 43 cSCC patients, and 15 non-lesional skin samples | miR-135b (up) | Cutaneous Squamous  Cell Carcinoma (cSCC) |
| (18) | miR‑135b inhibition could prevent tumorigenesis by inactivation of AKT/mTOR and JADE‑1 enhancement. | AKT/mTOR | JADE-1, Sox-2, Oct-4, Nanog, Aldh1, Slug, cleaved caspase3, and cleaved caspase9 | PANC-1, 293T | 9 pairs of PC tissue and ANCTs /mice | miR-135b (up) | Pancreatic Cancer (PC) |
| (19) | UCA1 as a sponge for miR-135a could lead to tumor promotion. | UCA1 | _ | SW1990, BxPC-3, MiaPaCa-2, PANC-1, CAPAN-1, HPDE | 50 pairs of PC tissues and ANCTs/mice | miR-135a (down) |  |
| (62) | miR-135 via targeting of PFK1 could inhibit aerobic glycolysis and promote tumor growth. | Glycolytic pathway, and p53 | PFK1, and cleaved caspase3 | MIA PaCa-2, PANC-1, BxPc-3, HT1080, MDA-MB-231, MCF-7, M229, 293T, and mouse PDAC cell lines | 9 pairs of PDAC tumors and ANCTs/ mice | miR-135 (up) | Pancreatic Ductal Adenocarcinoma (PDAC) |
| (63) | Over-expression of miR-135a could hamper cell proliferation and promote apoptosis via Bmi1 targeting. | PI3K/AKT | Bmi1, cyclin D1, Cdk2, Cdk4, p21, AKT, BAX, and Bcl-2 | PANC-1, BxPC-3, ASPC-1, HPDE6c7, HEK 293T | 11 pairs of PDAC tissues and ANCTs | miR-135a (down) |  |
| (64) | miR-135b inhibition could promote the osteogenic differentiation and bone formation. | _ | SMAD5, BSP, COLA1, OPN, and ALP | BM-derived mesenchymal stem cells, and HEK 293 | Bone marrow from 12 of MM patients and 7 healthy donors | miR-135b (up) | Multiple Myeloma (MM) |
| (65) | miR-135b inhibition could prevent cell proliferation. | _ | FOXO1, cyclin D1, p21, and p27 | C33A, HCC94, HeLa, HT-3, SiHa CaSKi, End1/E6E7 | _ | miR-135b (up) | Cervical Cancer  (CC) |
| (55) | miR-135b via activation by HSF1 could increase HCC cell migration, invasion and metastasis. | HSF1 | RECK and EVI5, MMP2, MMP9, and F-actin | HEK-293T, Huh-7, HepG2, SK-HEP-1, PLC/PRF/5, MHCC-LM3, SMMC-7721, Hep3B, MHCC97L | 120 pairs of HCC tissues and ANCTs/ mice | miR-135b (up) | Hepatocellular Carcinoma (HCC) |
| (66) | miR-135a activation via FVII/TF/PAR2 and then Atg14 targeting could inhibit autophagy and promote malignancy. | FVII/TF/  PAR2 | Atg14, LAMP-1, LC3A/B, cathepsin D | Hep3B, HepG2 | 103 pairs of HCC tissues and ANCTs | miR-135a (up) |  |
| (67) | Inhibition of miR‑135b or miR135a via AMOTL2 regulation could suppress cell viability, self‑renewal and stem cell marker expression. | Hippo pathway | AMOTL2 (miR-135a/b), STAT6, and GPX8 (miR-135a) | BTC, BTSC | 10 medulloblastoma tissues | miR-135a/b (up) | Medulloblastoma |

* Expression of this miRNA in tumoral tissues has been compared with its expression in normal/non-cancerous tissues of the same origin.

ANCT: adjacent non-cancerous tissue, IBD: inflammatory bowel disease, BLBC: basal-like breast cancer, QNBC: quintuple negative breast cancer, TMJ: temporomandibular joint, FFPE: formalin-fixed, paraffin-embedded
